# Tough and Resilient Hydrogels Enabled by a Multifunctional Initiating and Cross-Linking Agent

**DOI:** 10.3390/gels7040177

**Published:** 2021-10-21

**Authors:** Zhenxing Cao, Zhaoyang Yuan, Rui Wu, Haitao Wu, Biqiang Jin, Jing Zheng, Jinrong Wu

**Affiliations:** State Key Laboratory of Polymer Materials Engineering, College of Polymer Science and Engineering, Sichuan University, Chengdu 610065, China; caozx1201@163.com (Z.C.); zhaoyangyuan@stu.scu.edu.cn (Z.Y.); wurui641107971@stu.scu.edu.cn (R.W.); wht2281007108@163.com (H.W.); Elden_Jin@163.com (B.J.)

**Keywords:** hydrogels, toughness, resilience, entanglements, branched chains

## Abstract

Many high-strength hydrogels have been developed in recent years; however, few of them are both tough and resilient, and their intrinsic paradoxical nature makes designing a gel with both high toughness and high resilience a great challenge. To address this problem, we introduced both N,N,N,N-pentamethyldiethylenetriamine (PA) and N,N-methylenebisacrylamide (MBA) into polyacrylamide hydrogel networks to construct an entangled network that contains chemically cross-linked chains and branched chains simultaneously. The entanglements of branched chains can act as a physical cross-linking point to uniformly disperse stress on molecular chains, and chemical cross-linking ensures the stability of the hydrogel network. The increase in the number and length of branched chains is able to achieve an enhancement in strength while the slip of the entangled polymer chains can effectively achieve energy dissipation and can improve the toughness of the gel. Moreover, the resultant hydrogels exhibit an excellent resilience (>98%). Therefore, high toughness and resilience are achieved simultaneously. In addition, we also investigated the initiation mechanism of PA. This strategy creates a new way for the preparation of next-generation high toughness and high resilience hydrogel-based materials, which have promising applications in wearable, flexible strain/pressure sensors.

## 1. Introduction

As a sort of “soft and wet” material, hydrogels have been widely used in biomedical and other fields because of their tunable physicochemical properties and good biocompatibility [1]. However, conventional hydrogels usually exhibit poor mechanical properties, which severely limits some of their practical applications. To address this problem, many hydrogels with excellent mechanical properties have been designed over the past few decades, such as double network gels (DN) [2,3], nanocomposite gels (NC) [4], tetra-PEG gels [5,6], and slip-ring gels [7,8], the development of which began after the introduction of energy dissipation mechanisms. However, although some of the properties of these gels are far superior to those of conventional gels, they still do not demonstrate high toughness and elasticity synchronously. For instance, DN gels composed of two structurally asymmetric polymers show extremely high mechanical strength and tensile properties, but their resilience is usually low [9], while some gels exhibit better resilience but relatively low mechanical strength [10]. To address this problem, many efforts have been made in recent years [11,12,13,14,15,16]. Zhang et al. designed molecular springs by introducing helical peptide chains into the hydrogel network through peptide cross-linking agents, thus achieving hydrogels with both high toughness and high elasticity [17]. Kohzo Ito et al. ingeniously designed slip-ring hydrogels with strain-induced crystallization as the toughening mechanism, and the resulting hydrogels were able to recover nearly 100% more quickly under large mechanical stress [8]. It can be seen that despite the great efforts that have been made, the molecular structural design needed to achieve high toughness and high resilience hydrogels is still a great challenge for their use in long-term applications.

Another reason hydrogels lack mechanical strength is because of the low density of their polymer chains, which is due to the inhomogeneous swelling of hydrogels, and their loose structure weakens the spatial distance that is dependent on the interacting groups, thus reducing the energy dissipation efficiency [9,18]. Therefore, the general strategy that is used to achieve high toughness is the introduction of an energy dissipation mechanism, while for high elasticity, the homogeneity of the network needs to be improved. Unfortunately, highly ductile gels require the presence of large hysteresis loops in the stress–strain curve, while highly elastic gels require smaller energy dissipation [19]. As it can be seen, the toughness and elasticity for hydrogels are inherently contradictory, and there is a trade-off between them. Current energy dissipation is usually accomplished by the breakage of sacrificial bonds, including covalent [3], hydrogen [2,20], ionic [2,18,21], dynamic covalent bonds [22], and so on. However, these bonds are irreversible when broken, leading to large hysteresis loops, hence the low resilience of gels.

Inspired by the fact that the branched chains on the main chain of high-density polyethylene perform an important role on their properties [23,24,25], here, we propose that hydrogels with both high resilience and high toughness can be prepared by using the entanglements between the branched chains. The entanglements of branched chains can act as a physical cross-linking point, and the stress on the molecular chain is dispersed throughout the network, ensuring the integrity of the network while subtly introducing energy dissipation. In this work, acrylamide as a monomer, N,N,N,N′,N′-Pentamethyldiethylenetriamine (PA) combined with N,N-methylenebisacrylamide (MBA) to introduce few chemical cross-linking was chosen to design a dual cross-linked hydrogel (DC) with both chemical cross-linking and physical entanglements. When the gel is deformed, the slip of the entangled polymer chains can effectively dissipate energy, thus improving the toughness of the gel. Since there is also some chemical cross-linking, the slip is somewhat restricted, and the hysteresis loop shows a lower dissipation energy, thus realizing the resilience. The unique design of the molecular structure significantly endows the DC hydrogels with high toughness and high resilience. In addition, the strategy is also applicable to other systems, opening up new avenues for the preparation of a next-generation highly tough and elastic hydrogel-based materials, which exhibit promising applications in wearable, flexible strain/pressure sensors.

## 2. Results and Discussion

### 2.1. Initiation Mechanism of PA

**Figure 1:**
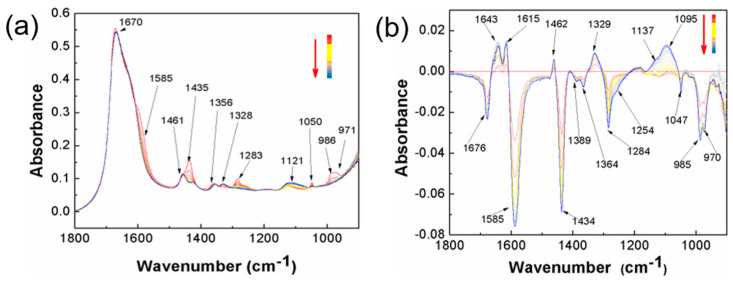
Real-time FT-IR spectrum and differential spectrum of the KPS-PA initiating systems. (**a**) FI-IR spectrum of KPS-PA; (**b**) differential spectrum of KPS-PA.

Inspired by the fact that polyamines can be used as a reductant in oxidation-reduction reactions, we selected a PA with polyfunctionality and potassium persulfate (KPS) as a redox initiating system for the preparation of high-performance hydrogels and investigated the initiation mechanisms. In order to observe the evolution of the groups in the PA more accurately, the initiation system of KPS-PA was constructed to initiate the polymerization of acrylamide, and it was compared with the KPS-NaHSO_3_ system constructed in our previous work [26], and the real-time dynamic IR spectra were traced. As shown in Figure 1, the absorption peaks with the same changes in both systems are at 1680 cm^−1^, 1588 cm^−1^, 1459 cm^−1^, 1435 cm^−1^, 1364–1388 cm^−1^, 1328 cm^−1^, 1282 cm^−1^, 1046–1053 cm^−1^, 985 cm^−1^, 970 cm^−1^, etc. The absorption peak for the stretching vibration of C=O at 1680 cm^−1^ is the amide I band; the C=C absorption peak is at 1588 cm^−1^, which decreases with the increase of the polymerization time. Additionally, superimposed here is the absorption peak of the amide II band, and the increase in absorbance at 1650–1617 cm^−1^ is precisely ascribed to the movement of the amide II band. The absorption peak at 1459 cm^−1^ is the variable angle vibration of the -CH_2_- on the polymer chains, which increases with the generation of the chains. The absorption peak of sulfate appears at 1095 cm^−1^. The absorption peak of the stretching vibration of C-N carried by the by-products in the reaction appears at 1282 cm^−1^, which is consumed as the reaction proceeds, so the peak gradually decreases.

**Figure 2:**
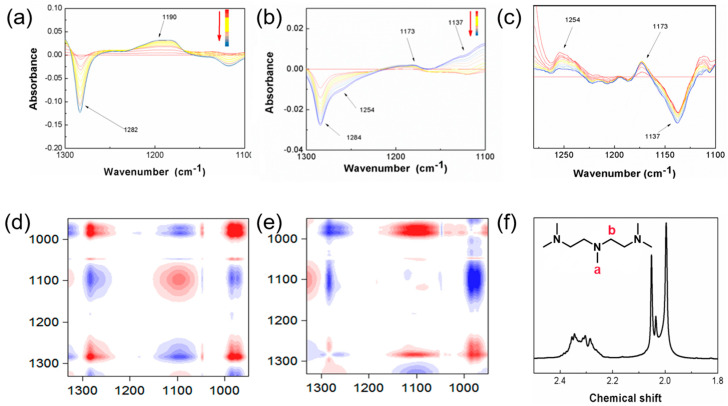
The differential spectrum of the two initiating systems (**a**) KPS-NaHSO_3_ and (**b**) KPS-PA at the wavelengths of 1300–1100 cm^−1^. (**c**) The derivative curves of differential spectrum of KPS-PA at the wavelengths of 1300–1100 cm^−1^. (**d**) Synchronous and (**e**) asynchronous 2D IR spectrum of the polymerization of AM initiated by KPS-PA. (**f**) ^1^H-NMR spectrum of pentamethylene triamine (PA).

Compared to the KPS-NaHSO_3_ system, the spectrum of the PA-initiated system shows a significant shoulder peak between 1280 cm^−1^ and 1090 cm^−1^ (Figure 2a,b). When the dynamic IR spectra are derived once (Figure 2c), the specific peak positions are found to be at 1254 and 1137 cm^−1^, respectively. The groups attached to the nitrogen atom of PA are -CH_3_ and -CH_2_-, both of which are electron-donating groups, causing the C-N stretching vibration absorption peak shift to lower wave numbers. When the -CH_3_ adjacent to the nitrogen atom loses hydrogen, the bending vibration peak of -CH_2_- forms at 1173 cm^−1^; conversely, the tertiary carbon stretching vibration peak forms at 1137 cm^−1^. As shown in Figure 2c, the peak at 1173 cm^−1^ is almost unchanged, while the peak at 1137 cm^−1^ is highly variable, indicating that the methylene of PA is involved in the redox reaction.

In order to further investigate the sequence of the reaction of the groups and to determine the active sites generated by the participation of PA in the reaction, we performed a two-dimensional infrared correlation spectrum analysis on the dynamic infrared spectrum of the KPS-PA system. As shown in Figure 2d,e, at 1254 cm^−1^ and 1137 cm^−1^, the synchronous correlation spectrum shows a negative correlation, while in the asynchronous graph, it shows a negative correlation, which can prove that the group reaction occurs synchronously and that the formation of tertiary carbon occurs simultaneously with the displacement of the carbon-nitrogen group [27]. In addition, the correlation spectra at both positions exhibit evidence that they are not uncorrelated, further proving that the methyl group adjacent to the nitrogen atom is not involved in the reaction. To sum up, we conclude that the methylene group in PA is involved in the redox reaction.

The above IR analysis demonstrates the changes that occur in the PA groups for the KPS-PA system, revealing the possibility that PA is capable of forming branched sites. To further confirm the existence of this structure, we first performed NMR characterization on PA. As shown in Figure 2f, there are mainly two types of active hydrogens present within PA, namely methyl a and methylene b, with chemical shifts corresponding to about 2.19~2.22 and 2.5 ppm, respectively.

**Figure 3:**
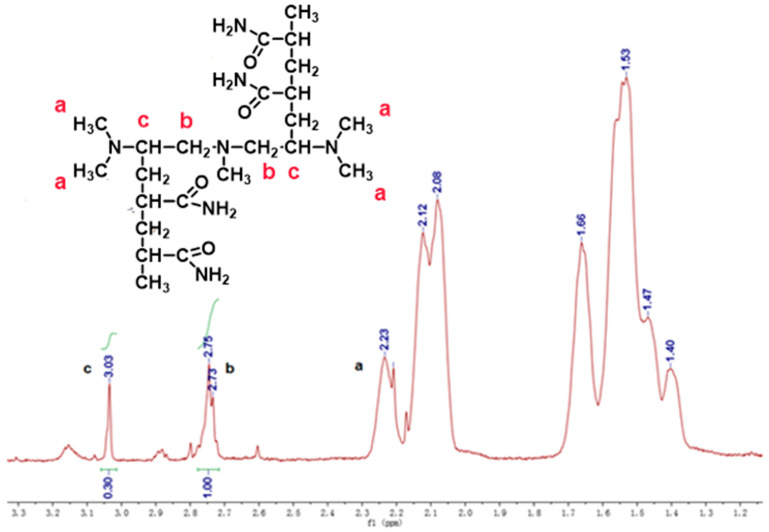
^1^H-NMR spectrum of the model polymers of PA initiated PAM.

The NMR spectra of the PA-initiated system are shown in Figure 3. The alternating methylene and methine on the chains appear in the peaks at around 1.5 and 2.1 ppm, respectively. The -CH_3_ (a) at the PA structural unit shows a peak at 2.2 ppm, and the original -CH_2_- (b) is involved in the redox reaction to form methine with a peak at 3.03 ppm compared to the theoretical value of 2.8 ppm on account of deshielding effect. The chemical shift of -CH_2_- (b) is also shifted to 2.7 ppm due to the same effect. From the integrated area of the peak, S(c):S(b) = 0.3:1, the most simplified ratio of CH:CH_2_ is 3:5, indicating that some of the methylene in PA is oxidized to form methine, which, in turn, initiates the polymerization of the acrylamide monomer at this location.

**Figure 4:**
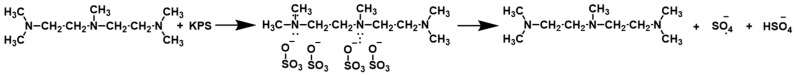
The initiation mechanism of PA and KPS.

Based on the above results, we obtained the initiation mechanism of PA and KPS as shown in Figure 4. Upon attack by persulfate, the methylene in PA removes a hydrogen to form a radical. All four methylenes in PA can remove hydrogen to form a radical, so it can be regarded as a tetrafunctional cross-linker. However, the value of CH:CH_2_ in the NMR spectrum is 3:5, proving that only some of the methylene groups form free radicals during polymerization.

### 2.2. Kinetics Study on PA Gels

The initiation mechanism of the PA/KPS system shows that when PA collides with KPS, the methylene group loses a proton to form a reactive radical, which initiates polymerization to form macromolecular chain radicals. During the polymerization process, these macromolecular radicals can form chemical cross-links by coupling termination or form branched chains by means of disproportionation termination [28,29]. Further, the branched chains can also form physical cross-linking sites in the network by intertwining. It is thus clear that PA is not only a reducing agent in this system but that it can also be seen as a cross-linking agent. Therefore, the initiation system can initiate the polymerization of acrylamide to form gels without additional cross-linking agents.

**Figure 5:**
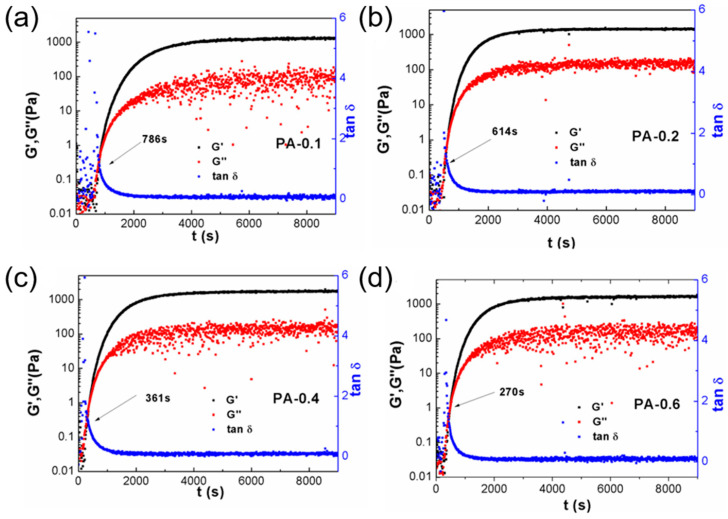
Evolution of the elastic modulus (G′), the viscous modulus (G″), and tanδ during the polymerization for different PA hydrogels of (**a**) PA-0.1, (**b**) PA-0.2, (**c**) PA-0.4, and (**d**) PA-0.6.

The gels prepared by the PA/KPS initiation system were called PA gels, and the gelation process of gels with different PA concentrations was traced online using a rheometer, as shown in Figure 5. It can be seen that all of the gels underwent the same reaction process. The elastic modulus (G′) and viscous modulus (G″) rise slowly and finally reach a plateau. The intersection of G′ and G″ can be regarded as the gelation point of the polymerization reaction, and the time of the gelation point can be used to measure the speed of the reaction. As the concentration of PA increases from 0.1% to 0.6%, the time required to reach the gel point decreases from 786 s to 270 s, and the increased polymerization reaction rate proves that PA is an initiator. After reaching the plateau, the G′ of the sample is much higher than G″, demonstrating the gel formation and indicating that PA is also a cross-linking agent.

### 2.3. Preparation of High-Performance DC Hydrogels

**Figure 6:**
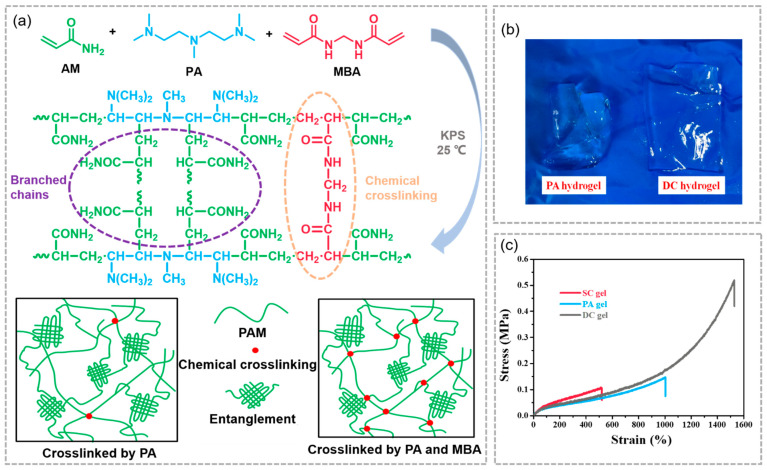
(**a**) The formation mechanism of the DC hydrogel; (**b**) the swollen PA and DC hydrogels; (**c**) Tensile stress–strain curves of the hydrogels of SC, PA and DCgels.

During the study, we found that the degree of chemical cross-linking in the gels that were only cross-linked by PA was weak. In order to compensate for the lack of chemical cross-linking points in the gel, MBA was added to introduce some additional chemical cross-linking points in the network. The gels prepared using this system are likewise hydrogels with both physical and chemical cross-linking and that exhibit high-performance and were called DC-x-y hydrogels. Moreover, hydrogels that are only cross-linked by PA were called PA-x gels, and hydrogels that were only cross-linked by MBA were called SC-y gels. The x and y represent the feeding amount of PA and MBA, respectively. A typical one-pot polymerization procedure and mechanism for the target hydrogels is illustrated in Figure 6a. It is clear that PA and SC gels are quite brittle and are easily broken after being soaked in urea solution (Figure 6b), and the distinct mechanical performances of the gels before and after the toughening process are characterized by tensile tests (Figure 6c). PA plays a role in the network, generating branched chains that can form physical cross-linking points in the gel through entanglements. In addition, the coupling termination of the branched chains and the introduction of MBA lead to the formation of a chemical cross-linked network in the gel.

### 2.4. Mechanical Measurements

**Figure 7:**
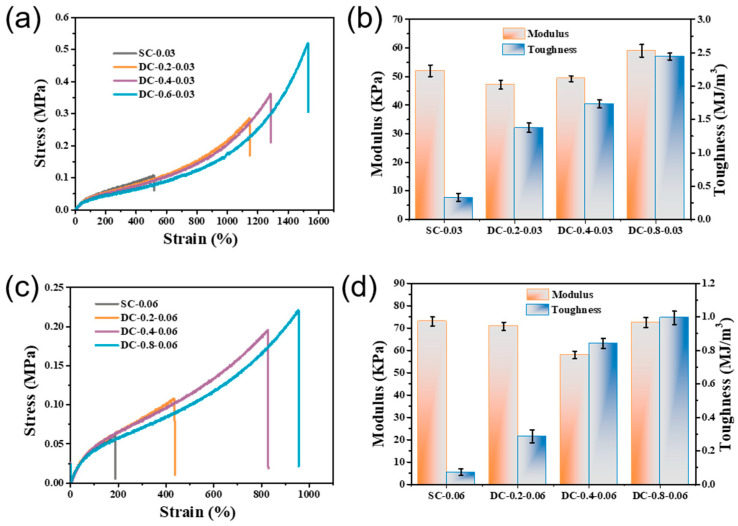
Tensile stress–strain curves (**a**,**c**) and mechanical properties (**b**,**d**) of hydrogels cross-linked with different concentrations of PA and MBA.

DC hydrogels are highly transparent and exhibit extraordinary flexibility and toughness, and their mechanical properties are influenced by the composition of the gel matrix and the concentration of PA and MBA. As shown in Figure 7a,c, the conventional SC gel is brittle, and the elongations of SC-0.03 and SC-0.06 are only 500% and 200%, respectively, and the toughness is less than 0.4 MJ/m^3^. When 0.2% PA is added, the elongation significantly increases to 1,100%, and the strength increases to 280 kPa. When the concentration of PA increases to 0.8%, the elongation increases to 1520%, and the strength can further increase to 0.5 MPa and can achieve an amazing toughness of 2.44 MJ/m^3^. It is clear that the introduction of PA can significantly improve the mechanical performance of the hydrogel.

**Figure 8:**
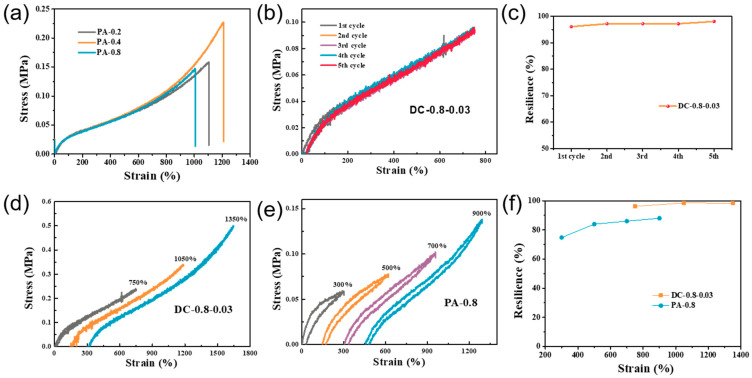
(**a**) Tensile stress–strain curves of PA gels; (**b**) five successive loading–unloading curves of DC-0.8-0.03 gels at the strain of 750% and (**c**) corresponding resilience. (**d**) Stress–strain curves of DC-0.8-0.03 gels during three consecutive loading–unloading cycles at maximum strains of 750%, 1050%, and 1350%. (**e**) Stress–strain curves of PA-0.8 gels during four consecutive loading–unloading cycles at maximum strains of 300%, 500%, 700%, and 900%. (**f**) Resilience of PA-0.8 gels and DC-0.8-0.03 gels.

Figure 7b,d reveal that the toughness of the DC gel is more sensitive to PA and MBA concentrations, while the modulus is not sensitive to the PA concentration but that it is sensitive to the MBA concentration. As the concentration of MBA increases, the strength increases but elongation decreases significantly, and the toughness of the gel becomes worse. We attributed this to the following two aspects: On the one hand, the increase of MBA accelerates the non-uniformity of the network. On the other hand, the chemical cross-linked network becomes tight, and the motion of the branched chains is restricted, weakening the ability of the hydrogel to dissipate this energy through untwisting. Therefore, in this study, we needed to keep the MBA at a low concentration. Interestingly, the PA gels consistently exhibited the same brittleness as the SC gels when the concentration of MBA reached 0.2%, regardless of the amount of PA that was added. For the PA gels, the elongation and toughness are greater than those of conventional gels (Figure 8a) but are far less than the DC gels.

### 2.5. Deformation Recoverability and Enhancement Mechanism

Although many high-toughness hydrogels have been reported in recent years, most of them sustain a low fatigue resilience. To investigate the fatigue resilience of the gels, five consecutive loading and unloading cycles were performed at 750% strain. As shown in Figure 8b, the stress–strain curves largely overlapped, especially after the second cycle. A smaller hysteresis circle was seen for each cycle. This is quite different from many of the hard hydrogels reported in the literature, where a large hysteresis loop is typically observed. Resilience is a measure of a material’s ability to deform reversibly and is calculated as the ratio of the energy recovered during unloading to the work done on the specimen during loading [17]. A resilience of 96.12% was found in the first loading-unloading cycle. This increased to 97.22% in the 2nd cycle and eventually reached 98.11% (Figure 8c).

**Figure 9:**
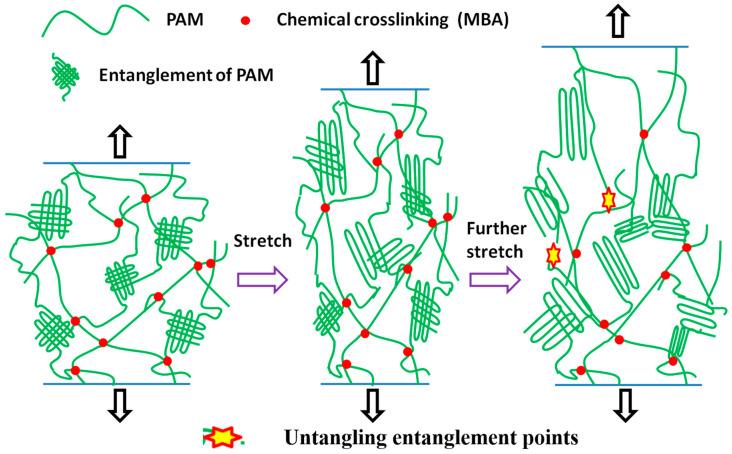
Schematic showing possible structural scenarios during network deformation in DC hydrogels.

Similarly, we completed stepwise cycles for the PA and DC hydrogels, respectively, to further illustrate that the entanglement network formed by the branched chains played an energy dissipation role in the gel. As shown in Figure 8d–f, the energy dissipation of the PA gel is more obvious, and the hysteresis loop at a small strain rate can be an order of magnitude higher than that of DC gel. Additionally, the hysteresis and loop residual strain are larger as the strain increases. Based on the above results, we obtained the toughening mechanism of the DC gels, as shown in Figure 9. The energy dissipation for DC gel is generated by the slip or untwisting of the branched chains, and although the slip or untwisting of the branched chains is affected by the external chemical cross-linking network, the energy dissipation mechanism introduced through the branched chains can lead to a significant enhancement of the toughness of the gel.

## 3. Conclusions

In conclusion, we demonstrate that synthetic hydrogels with both high toughness and high resilience can be designed by introducing polyamines into the hydrogel network. We also investigated the PA initiation mechanism by dynamic infrared and NMR and introduced both physical entanglements and chemical cross-linking junctions to prepare DC hydrogels, thus providing a new energy dissipation mechanism. The toughness and tensile properties of the peptide cross-linked gels were significantly improved compared to those of single MBA or PA cross-linked hydrogels. The resultant DC hydrogels exhibited a small hysteresis loop and thus had high resilience (98.5%). This strategy is universal and provides a new way to prepare highly elastic and tough gels.

## 4. Materials and Methods

### 4.1. Materials

Acrylamide (AM)was purchased from Chengdu Huaxia Chemical Reagent Co., Ltd., Chengdu, China. N,N-methylenebis (acrylamide) (MBA), N,N,N,N-Tetramethylethylenediamine (TEMED), and potassium persulfate (KPS) were obtained from Tianjin Bodi Chemical Industry Ltd., Tianjin, China. N,N,N,N′,N′-Pentamethyldiethylenetriamine (PA) was purchased from Chengdu Best Reagent Factory, Chengdu, China. All reagents were of analytical grade and were not further purified prior to use. In this study, deionized water was used to prepare the aqueous solutions.

### 4.2. Preparation of the DC (Dual Cross-Linked) Hydrogels

A typical DC hydrogel was synthesized in aqueous solution by in situ free radical polymerization. In brief, 30 g of AM monomer, a certain amount of PA, cross-linker MBA, and 40 mg of KPS were weighed and dissolved in deionized water. After complete dissolution, the solution was degassed by nitrogen for 10 min to remove dissolved oxygen. Finally, the solution was poured into a preformed mold with two glass plates (1 mm thick silica plates sandwiched between the plates) and were held for 24 h at room temperature to complete the reaction. The final resultant hydrogels were denoted as DC-x-y, where x and y represent the mass fraction (compared with AM) of PA and MBA, respectively.

### 4.3. Dynamic Infrared Characterization

After configuring the above precursor solution, about 2 mL was quickly aspirated and dropped into the CaF_2_ liquid cell and then transferred into Fourier transform infrared spectroscopy (FTIR, Nicolet 6700, New York, NY, USA) for real-time monitoring with a resolution of 1 cm^−1^.

### 4.4. Nuclear Magnetic Resonance (NMR) Characterization

The PA and hydrogel samples for NMR were prepared in D_2_O, and the structures were characterized by a Swiss Confederation Bruker AV600 NMR. The samples were prepared as follows: a certain proportion (low concentration) of AM, MBA, PA, and KPS were weighed and dissolved into solutions in D_2_O, respectively. After passing nitrogen and removing oxygen, the mixture was transferred to the NMR tube and tested after complete reaction.

### 4.5. Rheological Testing

All rheological measurements were performed using a rheometer (AR2000EX, TA Instrument Corp., Ontario, CA, USA) equipped with a flat plate (40 mm diameter) and a temperature control device. About 2 mL of fresh reaction solution was placed on the platform, and a stress of 0.1 Pa and a frequency of 1 Hz were selected for the time-scanning sweep to monitor the change of the modulus with the reaction time. During the measurement, a solvent trap was used to keep the sample hydrated.

### 4.6. Mechanical Measurements

The mechanical measurements of all of the hydrogel samples were performed using an Instron 5567 tensile tester (Instron Corporation, Boston, MA, USA). The thin film-like gel samples were cut into a dumbbell shape (Standard JISK6251-7, length of 35 mm, width of 2 mm, length of 12 mm) for testing. For the uniaxial tensile test, Young’s modulus and toughness were calculated from the initial slope and the integral area of the stress–strain curve, respectively. To investigate the deformation recoverability, two types of cyclic stretching were performed on the samples. For one, and the samples were stretched to the constant maximum strain (800%) and were then returned to the initial strain at the same rate successively four different times; the other was a step cycle with a maximum strain of 100% for the first time, and thereafter, the strain increased by 100% for each cycle until the maximum strain reached 500%. The tensile speed was 100 mm·min^−1^, and each sample was tested three times.

## Data Availability

The data presented in this study are available upon request from the corresponding author.

## Sample Availability

Samples of the compounds are available from the authors.
